# Aqueductal developmental venous anomaly as an unusual cause of congenital hydrocephalus: a case report and review of the literature

**DOI:** 10.1186/1752-1947-6-7

**Published:** 2012-01-11

**Authors:** David Paulson, Steven W Hwang, William E Whitehead, Daniel J Curry, Thomas G Luerssen, Andrew Jea

**Affiliations:** 1 Division of Pediatric Neurosurgery, Texas Children's Hospital, Department of Neurosurgery, Baylor College of Medicine, Houston, Texas. USA

## Abstract

**Introduction:**

Aqueductal stenosis may be caused by a number of etiologies including congenital stenosis, tumor, inflammation, and, very rarely, vascular malformation. However, aqueductal stenosis caused by a developmental venous anomaly presenting as congenital hydrocephalus is even more rare, and, to the best of our knowledge, has not yet been reported in the literature. In this study, we review the literature and report the first case of congenital hydrocephalus associated with aqueductal stenosis from a developmental venous anomaly.

**Case presentation:**

The patient is a three-day-old, African-American baby girl with a prenatal diagnosis of hydrocephalus. She presented with a full fontanelle, splayed sutures, and macrocephaly. Postnatal magnetic resonance imaging showed triventricular hydrocephalus, suggesting aqueductal stenosis. Examination of the T1-weighted sagittal magnetic resonance imaging enhanced with gadolinium revealed a developmental venous anomaly passing through the orifice of the aqueduct. We treated the patient with a ventriculoperitoneal shunt.

**Conclusions:**

Ten cases of aqueductal stenosis due to venous lesions have been reported and, although these venous angiomas and developmental venous anomalies are usually considered congenital lesions, all 10 cases became symptomatic as older children and adults. Our case is the first in which aqueductal stenosis caused by a developmental venous anomaly presents as congenital hydrocephalus. We hope adding to the literature will improve understanding of this very uncommon cause of hydrocephalus and, therefore, will aid in treatment.

## Introduction

It is common for intraventricular cerebrospinal fluid (CSF) flow to become obstructed at the aqueduct of Sylvius [1]. The obstruction may be caused by a tumor, congenital etiology, or post-inflammatory gliotic atresia, among other conditions [1-3]. Obstruction by a vascular malformation at the aqueduct is a very rare cause [2,4-7], and aqueductal stenosis attributable to a developmental venous anomaly (DVA) is perhaps the most uncommon [8].

Although DVAs are thought of as congenital findings, all 10 previously reported cases became symptomatic as older children or adults. To the best of our knowledge, we present the first case of symptomatic congenital hydrocephalus from aqueductal obstruction due to a DVA.

## Case presentation

**Figure 1:**
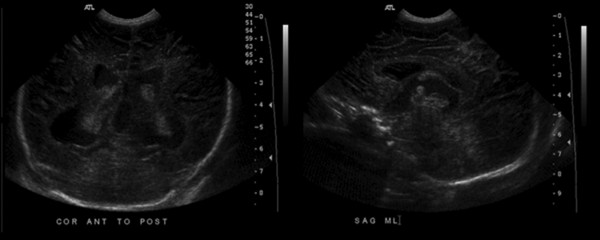
**Prenatal ultrasound at 37 weeks gestation shows triventricular hydrocephalus, suggesting obstruction at the aqueduct of Sylvius**.

The patient transferred to our institution was a three-day-old, African-American baby girl with a diagnosis of congenital hydrocephalus. Prenatal ultrasonographic screening at 20 weeks was unremarkable. At 37 weeks, the maternal abdominal girth exceeded the expected range, and a second ultrasound revealed interval development of congenital hydrocephalus (Figure 1). A Caesarian section was planned, but the mother went into spontaneous labor, and the baby was delivered vaginally before the procedure could be performed.

The baby was born healthy with no obviously anomalous anatomy aside from macrocephaly, splaying of her cranial sutures, and a full anterior fontanelle. She opened her eyes spontaneously and cried to stimulation. Her pupils were equal and reactive to light; she had normal muscle tone and was moving all extremities symmetrically. Her head circumference was 35.5 cm, which correlated to the 50th percentile.

**Figure 2:**
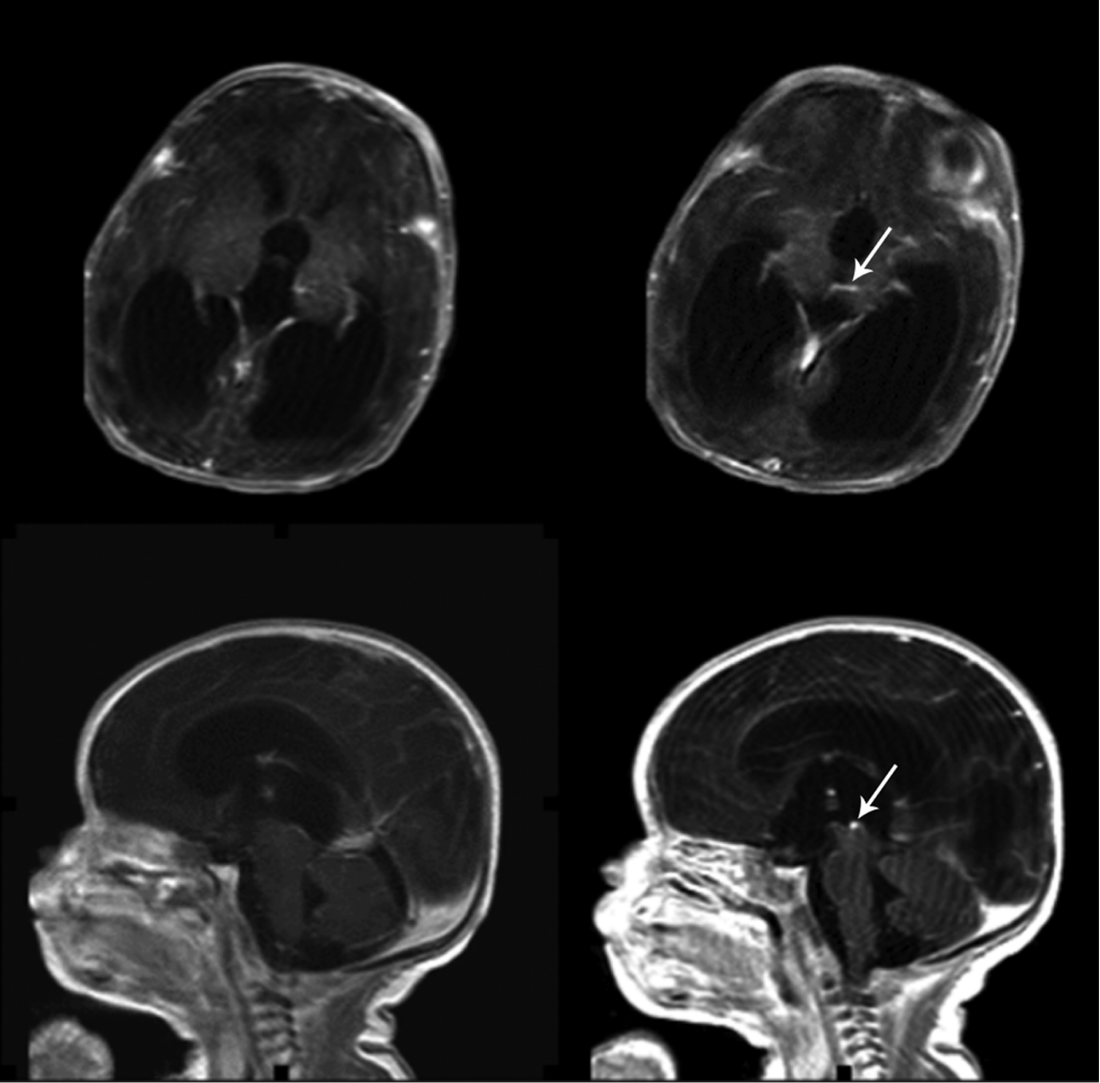
**Postnatal T1-weighted axial and sagittal MRI with gadolinium at three days of life demonstrates a patent aqueduct of Sylvius, but a developmental venous anomaly (white arrow) extending from the left thalamus converging on a central draining vein bridges across the proximal orifice of the aqueduct of Sylvius and obstructs the aqueduct**.

An MRI of the brain corroborated hydrocephalus with the presence of a DVA within the third ventricle (Figure 2). Even after re-reviewing the MRI of the brain with contrast with our neuroradiologist, we found that the DVA was intraventricular (inside the posterior third ventricle) in the region of the aqueduct rather than the quadrigeminal cistern. Additionally, the vein of Galen was normal size for the patient's age, and we did not see evidence of venous congestion in other regions of the brain which goes against the notion that the dilated vein seen in the third ventricle was secondary to congestion from hydrocephalus. We also closely examined the MRI of the brain with contrast with our neuroradiologist and found that the MRI did not suggest any vascular malformation. T2-weighted images did not demonstrate any abnormal signal flow voids. We did not feel that more invasive investigation with cerebral angiography and all its attendant risks was warranted in this case.

The baby's head circumference gradually increased over several days, and serial ultrasound examination showed she had progressively worsening ventriculomegaly, so she underwent placement of a ventriculoperitoneal shunt on her fifth day of life.

She was positioned supine with the head turned left, exposing the right occipital scalp. A curvilinear skin incision over the occipital scalp centered on the lambdoid suture was made for ventricular access. A linear skin incision in the midline periumbilical area was made to access the peritoneum for the distal end of the shunt catheter. A PS Medical low-pressure valve and distal catheter was then tunneled and passed in a typical fashion.

Ultrasound was used to define the trajectory of the ventricular catheter [9]. Xanthochromic CSF was visualized from the ventricular catheter corroborating old blood that was identified on the preoperative MRI. The shunt was then connected and placed in the peritoneum. The baby tolerated the procedure without incident.

She recovered well from the surgery and did not experience any post-operative complications. Routine post-operative imaging identified proper positioning of the shunt components and interval improvement of the hydrocephalus. Clinically, the patient recovered from the procedure and was subsequently discharged home in good condition.

## Discussion

A number of congenital and acquired factors are known to cause aqueductal stenosis and subsequent obstructive hydrocephalus [1-3]. Vascular lesions causing aqueductal stenosis are rare but, when present, can be attributed to a DVA (or venous angioma), vein of Galen malformation, abnormal draining vein, ectatic basilar artery, dural arteriovenous fistula, arteriovenous malformation, or cavernoma [2,3,5-8,10-15].

Aqueductal stenosis caused by a vein or DVA is extremely rare [3-5,10,15-17]. Only 10 cases have been reported (Table 1) in our review of the literature. There were 6 females and 4 males. Most patients were older children or young adults, with a mean age of 29 years (range 7 to 58 years). Symptoms were present for one month to several years prior to diagnosis and treatment. Headache was the most common presenting symptom. In our opinion, what makes our case unusual and worthy of publication is that our patient would be the youngest reported case in the literature with hydrocephalus occurring in the perinatal period from DVA.

**Table 1:** Patients with hydrocephalus from aqueductal stenosis from venous lesions (modified from Giannetti *et al*.)

Author, Year [Reference]	Age/Sex	Symptoms (duration)	Imaging	Venous lesion	Treatment
Rosenheck, 1937 [16]	58 yo/F	Mental deterioration (5 years)	Postmortem	DVA	None

Avman and Dincer, 1980 [10]	35 yo/F	Headache (7 years)	Ventriculography, CT, and angiography	Venous element	Stent

Watanabe *et al*., 1991 [3]	39 yo/M	Headache (1 year)	CT, MRI, and angiography	Venous malformation	Shunt

Oka *et al*., 1993 [7]	43 yo/F	Seizures (2 months)	CT, MRI, and angiography	Venous malformation	ETV

Blackmore and Mamourian, 1996 [5]	16 yo/F	Headache, behavior changes (2 months)	MRI	DVA	None

Bannur *et al*., 2002 [4]	11 yo/M	Headache (5 months)	CT and MRI	DVA	Shunt

Sato *et al*., 2004 [2]	28 yo/F	Headache, diplopia (N/A)	CT, MRI, and angiography	DVA	ETV

Yagmurlu et al., 2005 [15]	7 yo/F	Headache (1 month)	MRI	DVA	None

Giannetti *et al*., 2008 [8]	42 yo/M	Headache, behavior changes (1 year)	CT and MRI	DVA	ETV
	
	18 yo/M	Headache (6 years)	CT and MRI	Venous loop	ETV

Present case	Birth/F	Full fontanelle, splayed sutures, increased head circumference	MRI	DVA	Shunt

CT, computed tomography; DVA, developmental venous anomaly; ETV, endoscopic third ventriculostomy

In our case and a recent report of two other cases, an MRI based on a gadolinium-enhanced T1-weighted sequence revealed the aqueductal obstruction and its causes [8]. Aqueductal stenosis may be confirmed during endoscopic surgery [8], but direct visualization is not necessary. Likewise, invasive angiography is not necessary for the diagnosis of these venous lesions [4,8,18,19]. Unfortunately, we do not have an magnetic resonance angiography (MRA) or magnetic resonance ventriculography (MRV) to add to our case report. We did not initially suspect a venous anomaly as a possible cause for hydrocephalus, so we did not order an MRA or MRV upfront. Moreover, we do not feel that the risks justify putting the baby through general anesthesia again to obtain an imaging study that would confirm what is already seen on her MRI of the brain with contrast - a DVA causing obstructive hydrocephalus at the level of the origin of the cerebral aqueduct.

DVAs are usually benign lesions and considered normal variants that drain healthy brain tissue and do not need treatment because there is no risk of rupture and bleeding. Attempts to remove or occlude them may cause venous infarction or edema of normal parenchyma [4,5,15]. Therefore, primary treatment should be targeted towards addressing hydrocephalus, if the DVAs are symptomatic. Treatment options in these cases include close observation, aqueductal stenting, shunt placement, and endoscopic third ventriculostomy (Table 1).

A discussion of the pros and cons of endoscopic third ventriculostomy (ETV) versus ventriculoperitoneal shunt (VPS) to address hydrocephalus is beyond the scope of this report. In brief, we chose to treat our patient with a VPS because of ETV's high rate of failure in the infant age group [20].

## Conclusions

Aqueductal stenosis and consequent obstructive hydrocephalus by a DVA is extremely rare. Congenital hydrocephalus can present in this way. Enhanced T1-weighted sagittal MRI sequences may be informative for examination of the aqueductal region. No treatment is recommended for the DVA; however, CSF diversion may be necessary for the treatment of hydrocephalus. ETV should be considered in older children with this pathology or later shunt malfunction in this population.

### Consent

Written informed consent was obtained from the patient's next of kin for publication of this case report and any accompanying images. A copy of the written consent is available for review by the Editor-in-Chief of this journal.

## Competing interests

The authors declare that they have no competing interests.

## Authors' contributions

DP and SH were major contributors in writing and editing the manuscript. WEW, DJC, and TGL provided critical revisions. AJ was a major contributor in the design, writing, and editing of the manuscript. All authors read and approved the final manuscript.
